# Lab-on-a-Chip-Based PCR-RFLP Assay for the Detection of Malayan Box Turtle (*Cuora amboinensis*) in the Food Chain and Traditional Chinese Medicines

**DOI:** 10.1371/journal.pone.0163436

**Published:** 2016-10-07

**Authors:** Asing, Md. Eaqub Ali, Sharifah Bee Abd Hamid, M. A. Motalib Hossain, Shuhaimi Mustafa, Md. Abdul Kader, I. S. M. Zaidul

**Affiliations:** 1 Nanotechnology and Catalysis Research Center (NANOCAT), University of Malaya, Kuala Lumpur, Malaysia; 2 Centre for Research in Biotechnology for Agriculture (CEBAR), University of Malaya, Kuala Lumpur, Malaysia; 3 Institute of Halal Products Research, University of Putra Malaysia, UPM Serdang, Selangor, Malaysia; 4 School of Aquaculture and Fisheries, University of Malaysia Terrenganu, Kuala Terrenganu, Terrenganu, Malaysia; 5 Department of Pharmaceutical Technology, Faculty of Pharmacy, International Islamic University, Kuantan, Pahang, Malaysia; Mediterranean Agronomic Institute of Chania, GREECE

## Abstract

The Malayan box turtle (*Cuora amboinensis*) (MBT) is a vulnerable and protected turtle species, but it is a lucrative item in the illegal wildlife trade because of its great appeal as an exotic food item and in traditional medicine. Although several polymerase chain reaction (PCR) assays to identify MBT by various routes have been documented, their applicability for forensic authentication remains inconclusive due to the long length of the amplicon targets, which are easily broken down by natural decomposition, environmental stresses or physiochemical treatments during food processing. To address this research gap, we developed, for the first time, a species-specific PCR-restriction fragment length polymorphism (RFLP) assay with a very short target length (120 bp) to detect MBT in the food chain; this authentication ensured better security and reliability through molecular fingerprints. The PCR-amplified product was digested with *Bfa1* endonuclease, and distinctive restriction fingerprints (72, 43 and 5 bp) for MBT were found upon separation in a microfluidic chip-based automated electrophoresis system, which enhances the resolution of short oligos. The chances of any false negative identifications were eliminated through the use of a universal endogenous control for eukaryotes, and the limit of detection was 0.0001 ng DNA or 0.01% of the meat under admixed states. Finally, the optimized PCR-RFLP assay was validated for the screening of raw and processed commercial meatballs, burgers and frankfurters, which are very popular in most countries. The optimized PCR-RFLP assay was further used to screen MBT materials in 153 traditional Chinese medicines of 17 different brands and 62 of them were found MBT positive; wherein the ingredients were not declared in product labels. Overall, the novel assay demonstrated sufficient merit for use in any forensic and/or archaeological authentication of MBT, even under a state of decomposition.

## Introduction

Farm-to-fork food safety and quality has long been a goal, but to ensure it, both regulatory and market monitoring measures must be transparent across the globe [1]. Market surveys have revealed that 19.4% of all foodstuffs in the USA, 22% in Turkey and 8%in the UK are falsely labeled [2]. Furthermore, the recent entry of some alien species, such as rat meat, into the food chain [3] is highly alarming for public health, religious faith and the fair-trade economy, and the illegal trade of certain wild and endangered species especially threaten biodiversity, ecology and food safety [4]. The belief in certain purported health benefits such as the distinctive flavor, high protein content, low fat and cholesterol contents and the absence of health-threatening anabolic steroids in bush meat have continued to encourage the overhunting of wild species [5, 6]. The bones, shells, skins and eggs of certain endangered species, including turtles and tortoises, are believed to possess active healing attributes and invigorating elements, such as high contents of proteins and calcium [7, 8]. As a result, the market demand for these products has greatly surpassed their natural availability, and the restriction of their legal trade has prompted their turnover to hidden markets. According to the Convention on International Trade in Endangered Species (CITES) of Wild Fauna and Flora, the value of the illegal trade in wildlife was US $5–20 billion per year in 2007[9], and expert reports on international and internal security and illicit economies have revealed that approximately US $8–10 billion of the annual trade in protected species happens in Southeast Asia alone [10, 11].

According to the International Union for the Conservation of Nature (IUCN), of the 47,677 species that have been assessed, 17,300 are threatened; among these, one-fifth are mammals, one-third are amphibians, one-fourth are reptiles and 1,223 are birds[12]. Turtle species fall under the reptile umbrella, and there are approximately 460 varieties of freshwater turtles and tortoises around the globe [13]. Currently, all are enlisted under the most vulnerable clades of vertebrates on earth [14], and out of 293 IUCN Red-Listed freshwater turtles and tortoises, 88 species are found in Asia. According to Fund (2002), 3% of the world’s turtle species are already extinct, 9% are critically threatened, 18% are threatened, and 2% are at high risk in various habitats [13]. Among the Asian turtles, 1% are already extinct, 20% are critically endangered, 31% are endangered, and 25% are vulnerable. The Malayan box turtle (MBT) is the most common hard-shelled chelonian turtle species in Asia, and it is extensively distributed throughout habitats in Malaysia, Indonesia, India, Bangladesh, Thailand, Myanmar, Vietnam, Philippines, Singapore, Laos and Cambodia [15]. This species belongs to the *Cuora* genus, which encompasses a total of 12 turtle species, all of which are found in habitats in different geographical locations across the Asian peninsulas [14].

Recently, all of the *Cuora* species have been categorized as most vulnerable by the IUCN and listed in Appendix II of the CITES database[15]. Both the meat and shells of the *Cuora* genus are in high demand in international markets because of their uses in tonics and food as well as in antipyretic, analgesic and invigorating Chinese medicines [16]. Some researchers have found that turtle shells exert pharmacological effects against several diseases including hepatic and stress-related ailments [17], cancer [18], and immunomodulation [19]. Each year, more than 10 million live Asian box turtles (*Cuora*) (ABT) are imported into southern China from Southeast Asian countries [13], and the Taiwan statistical report revealed that more than 120 metric tons of turtle shells were imported between 1992 and 1998 from mainland China [20]. These statistics indicate that overhunting of *Cuora* species has been rampant in Asia. The recent newspaper report of turtle egg consumption by a Malaysian Minister is highly alarming because it represents a great threat to nesting resources [21]. The enormous illegal trade cannot be sustained and it has already contributed to a series of collapses of regional turtle stocks in several countries [13].

Since 2005, the Malaysian government and the Department of Wildlife and National Parks (PERHILITAN) of Malaysia have jointly banned the export of MBT and other turtle species to other countries. However, the confiscation of 4.3 metric tons of reptiles, including lizards, snakes, freshwater turtles and tortoises, at the Thailand-Malaysia border area by the Malaysian customs department in 2010 was a strong piece of evidence of the black market trade in turtle and other reptile species [22]. Among another encounters, the Malaysian police department seized 10,000 turtle eggs in Sabah in 2008 [23], and in 2013, the Filipino police arrested two Malaysian poachers on Mangsee Island that were trafficking endangered turtle eggs into the Philippines from Malaysia [24].

Regarding health concerns, turtle and tortoise handling and consumption are not risk free because these animals are natural scavengers of waste materials and hosts of several microbes and heavy metals. The health risks associated with the consumption of or contact with turtle meat, eggs and shells include infections caused by bacteria (such as *Salmonella spp*. and *Vibrio spp*.), parasites (such as Spirometra, Trichinella, Gnathostoma, and Pentastomids), and various type of biotoxins such as lyngbyatoxins, cyanotoxins, cytotoxins, haemotoxins, mycotoxins, and neurotoxins [25]. Furthermore, it is a sensitive social and religious issue because the consumption of turtle-derived materials is prohibited by certain religions such as Islam [26]. Therefore, there is a definite need for a reliable authentication method to detect turtle species in the food chain and traditional multicure medicinal products; we believe this would either prevent or reduce illegal trade and the chance of adulteration by or substitution of turtle ingredients in common food, thus mitigating the health hazards caused by turtle-borne zoonoses and allergens[11].

Current species identification schemes are mainly based on DNA analysis due to some of its inherent features, such as the universal information content and excellent stability of the DNA molecule itself [27]. Protein-based analytical schemes including electrophoretic [28], chromatographic[29], spectroscopic[30], and immunological[31] approaches have lost their appeal because protein-based biomarkers are susceptible to denaturation under food-processing conditions [32], and both the type and content of lipid biomarkers could also be significantly changed through cooking practices[33]. In contrast, short-length DNA biomarkers are extremely stable even in degraded specimens, and their sequences are conserved within all of the tissues of an individual [2]. Consequently, DNA-based approaches such as species-specific PCR [34, 35], multiplex PCR [36, 37], PCR product sequencing [20, 38], PCR-restriction fragment length polymorphism (PCR-RFLP)[39], random amplified polymorphic DNA (RAPD) [40], DNA microsatellites,[41, 42], real-time PCR[35, 43] and DNA barcoding [44] have evolved as the methods of choice for fraud detection in food chain.

**Fig 1:**
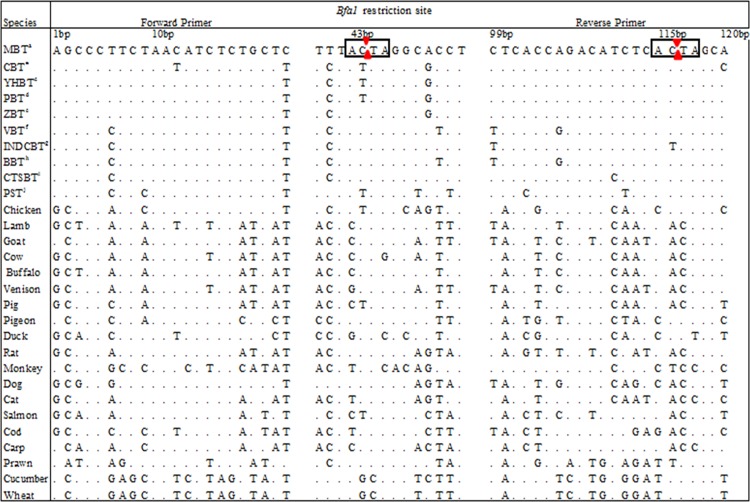
*In silico* analysis of the MBT-specific primers against 20 study species including 8 species of the *Cuora* genus with *Bfa1*-restriction sites. ^*a*^*Cuora amboinensis*-Malayan box turtle (MBT), ^b^*Cuora flavomarginata*-Chinese box turtle (CBT), ^c^*Cuora aurocapitata*-Yellow-headed box turtle (YHBT), ^d^*Cuora pani*-Pan’s box turtle (PBT), ^e^*Cuora zhoui*-Zhou's box turtle (ZBT), ^f^*Cuora picturata*-Vietnamese box turtle (VBT), ^g^*Cuora galbinifrons*-Indochinese box turtle (INDCBT), ^h^*Cuora bourreti*-Bourret’s box turtle (BBT), ^i^*Cuora trifasciata*-Chinese three-striped box turtle (CTSBT), and ^j^*Trachemys scripta-*pond slider turtle (PST).

Among the DNA-based methods, species-specific PCR-RFLP assays are highly reliable because they offer options for double-checking by first amplifying the specific target from a pool of complex matrices using species-specific primers[43] and, secondly, by authenticating the amplified PCR product through restriction fingerprints [39]. They are of special interest in meat identification because they exploit the sequence variation signatures that exist within a defined region of target DNA, allowing differentiation of even very closely related species through the digestion of selected DNA fragments using appropriate restriction enzymes[39]. PCR-RFLP assays have been documented as being able to distinguish between species as closely related as cattle-buffalo and sheep-goat [45], deer-cattle-sheep [46], various fish species[47], cattle-yak [48], and the eggs of different sea turtles[49]. Recently, species-specific PCR and PCR–DNA sequencing[20], PCR-RFLP[49], and quantitative PCR assays[35], have been used in the detection of MBT (*C*. *amboinensis*) and other turtle species, but most of these assays are based on longer-targets that break down during food processing[26, 39]. Here, for the first time, we described a very short-amplicon-length (120 bp) PCR-RFLP assay targeting the cytochrome b (cytb) gene for the confirmed identification of MBT in the food chain; the digestion of the PCR product (120 bp) by *Bf1* restriction endonuclease produced distinctive fingerprints (72 bp, 43 bp and 5 bp fragments) for MBT (Fig 1). We also constructed a phylogenetic tree using the MBT-specific 120-bp site of the cytochrome b gene against 66 reptile species, of which 44 were turtle and tortoise, 10 snake, 8 crocodile and 5 lizard species, to study the minimum and maximum distances between the target and non-target species and the theoretical likelihood of potential cross-species detection.

## Materials and Methods

### Sample collection and meat preparation

Ethical clearance was obtained from the University of Malaya’s Ethical Committee for Laboratory Animals as well as the Department of Wildlife and National Parks of Malaysia (PERHILITAN), located at Cheras in Malaysia, to study the occurrence of MBT meat in the food chain. Application was made to study 22 turtle and tortoise species including 12 species of the *Cuora* genus, but the Department of Wildlife and National Parks of Malaysia (PERHILITAN) only permitted the study of 5 species, namely, the Malayan box turtle (*Cuora amboinensis*), the pond slider turtle (*Trachemys scripta*), the Malayan soft-shell turtle *(Dogonia suplana)*, the yellow-headed temple turtle (*Heosemys annandalii*) and the elongated tortoise (*Indotestudo elongate)*. Authentic raw meat samples of Malayan box turtle and pond slider turtle and common meat species (chicken (*Gallus gallus*), cow (*Bos taurus*), goat (*Capra hircus*), pig (S*us scrofa domestica*), pigeon (*Columba livia*), sheep (*Ovis aries*), duck (*Anas platyrhychos*), buffalo (*Bubalus bubalis*)), fish (giant river prawn (*Macrobrachium rosenbergii*), Atlantic cod (*Gadus morhua*), salmon (*Salmo salar*), carp (*Cyprinus carpio*)) and plants (wheat (*Triticum aestivum*) and cucumber (*Cucumis sativus*)) were purchased from Pasar Borong, Pudu Raya and Selangor on three different days to increase the genetic diversity of the samples. Venison (*Odocoileus virginianus*) meats from three different animals were obtained in triplicate from the Faculty of Veterinary Science, University of Putra Malaysia located at Serdang in Selangor, Malaysia. Stray dog *(Canis lupus familiaris)*, cat (*Felis catus*) and rat (*Rattus rattus*) muscle meats were donated by Kuala Lumpur City Hall (KLCH) or (Dewan Bandaraya Kuala Lumpur (DBKL)), Air Panas, Kuala Lumpur and monkey (*Macaca fascicularis sp)* meat was a gift from the Department of Wildlife and National Park Malaysia (PERHILITAN/DWNPM), Cheras, Kuala Lumpur. DBKL routinely killed these animals for population control and public security in the town area and sufficient amount of muscle tissue samples of these species were taken from them following institutional and country laws. Information of all collected samples is compiled in Table 1. The identities of all of the collected samples of animal, fish and plant species were authenticated by veterinary, fisheries and botanical taxonomy experts and confirmed by sequencing (data not shown). All samples were transported under ice-chilled conditions and cut into small pieces for storage in a freezer at -20°C until further use to prevent the further degradation of the tissues and DNA. On the other hand, 153 medicinal samples of 17 different brands were collected from 5 different shops of Traditional Chinese medicines across Kuala Lumpur and Selangor.

**Table 1:** Information of collected food samples.

No	Species	Sources	Geographic coordinates of the sources	Animal Sources	Sample	Number of samples
1	Malayan box turtle	Wet market	Paser Borong, Pudu Raya and Selangor, Malaysia	Dead	Meat	30
2	Pond slider turtle	Wet market	Paser Borong, Pudu Raya and Selangor, Malaysia	Dead	Meat	15
3	Chicken	Tesco supermarket	Paser Borong, Pudu Raya and Selangor, Malaysia	Dead	Meat	30
4	Cow	AEON supermarket	Paser Borong, Pudu Raya and Selangor, Malaysia	Dead	Meat	30
5	Goat	AEON supermarket	Paser Borong, Pudu Raya and Selangor, Malaysia	Dead	Meat	30
6	Pig	Tesco supermarket	Paser Borong, Pudu Raya and Selangor, Malaysia	Dead	Meat	25
7	Pigeon	Wet market	Paser Borong, Pudu Raya and Selangor, Malaysia	Dead	Meat	20
8	Sheep	AEON supermarket	Paser Borong, Pudu Raya and Selangor, Malaysia	Dead	Meat	20
9	Duck	Wet market	Paser Borong, Pudu Raya and Selangor, Malaysia	Dead	Meat	25
10	Buffalo	Wet market	Paser Borong, Pudu Raya and Selangor, Malaysia	Dead	Meat	20
11	Prawn	Wet market	Paser Borong, Pudu Raya and Selangor, Malaysia	Dead	Meat	30
12	Cod fish	Wet market	Paser Borong, Kuala Lumpur, Malaysia	Dead	Meat	25
13	Salmon fish	AEON supermarket	Paser Borong, Pudu Raya and Selangor, Malaysia	Dead	Meat	25
14	Carp fish	Wet market	Paser Borong, Pudu Raya and Selangor, Malaysia	Dead	Meat	25
15	Wheat	Wet market	Paser Borong, Pudu Raya and Selangor, Malaysia		Powder	30
16	Cucumber	Wet market	Paser Borong, Pudu Raya and Selangor, Malaysia		Fresh vegetable	20
17	Dog	Dewan Bandaraya Kuala Lumpur (DBKL)	Kuala Lumpur, Malaysia	Dead	Meat	15
18	Cat meat	Dewan Bandaraya Kuala Lumpur (DBKL)	Kuala Lumpur, Malaysia	Dead	Meat	15
19	Rat meat	Dewan Bandaraya Kuala Lumpur (DBKL)	Kuala Lumpur, Malaysia	Dead	Meat	15
20	Venison	Veterinary Department	Kuala Lumpur, Malaysia	Dead	Meat	15
21	Monkey meat	Wildlife and National Parks (DWNP)	Cheras, Kuala Lumpur, Peninsular Malaysia,	Dead	Meat	15

### Design of oligonucleotide primers

Oligonucleotide primers specific to the MBT cytochrome b gene sequence were designed following a standardized procedure published in our earlier report [26]. The designed primers were Forward-5′-AGCCCTTCTAACATCTCTGCTC-3′, and Reverse-5′—CTCACCAGACATCTCACTAGCA-3′, which targeted a 120-bp site of the cyt b gene, and their specificity was ensured by three different testing systems [50]. Firstly, the basic local alignment algorithm search tool (BLAST) against non-redundant nucleotide sequences in the NCBI database identified the target species as well as the dissimilarity index value with other species. Secondly, the primers were aligned against 28 different non-target species, among which 20 were used for the experimental probing of cross-specificity, and 8 were *Cuora* species that were used to establish a relationship index among the most closely related species. The sequence of the closely related species, such as the 8 *Cuora* species, were aligned using the MEGA 5 software to identify the specific conserved and variable sequences to study the presence of mismatched bases. The designed primers were purchased from 1^st^ Base Laboratories Sdn. Bhd., Selangor, Malaysia. Finally, MBT-specificity was confirmed in a practical PCR experiment through a cross-amplification reaction in the presence of the target and 20 different non-target species cited above[50]. An internal positive control was used to amplify a 141-bp site of the 18S rRNA gene using previously reported universal primers for eukaryotes (EuF: 5′-GGTAGTGACGAAAAATAACAATACAGGAC-3′ and EuR: 5′-ATACGCTAT TGGAGC TGGAATTACC-3′ [51].

### Preparation of binary and ternary meat mixtures

To simulate the complexity of the matrices, two types of binary mixtures, i.e., MBT-beef and MBT-goat, were made in a total volume of 100 g of specimen by mixing MBT meat in a proportion of 10%, 1%, 0.1% and 0.01% with an adjusted amount of beef and goat meat[26, 39, 52]. A ternary admixture composed of MBT meat, chicken and wheat flour was made by mixing 10%, 1%, 0.1% and 0.01% of turtle meat into chicken and wheat flour, in which the MBT:chicken:wheat flower contribution was 20:80:100, 2:98:100, 0.2:99.8:100 and 0.02:99.98:100, respectively[52]. Finally, the required amount of deionized water was added to the admixtures and briskly ground with a blender (Pensonic Super Blender- PB-3205, 13600 Prai, Penang Malaysia) to obtain a homogenous, semi-solid slurry. To prevent contamination, each of the mixtures was made and tested on separate occasions in triplicate, and all of the prepared admixtures were stored at -20°C prior to DNA extraction.

### Collection and preparation of meat products

A total of 189 samples (21 X 9) of 21 different branded commercial meat products (Table 2), namely, chicken and beef meatball, burger and frankfurter products were cross-tested against the MBT-specific primers in triplicate. Dummy chicken and beef meatball, burger and frankfurter products were prepared following Ali et al. (2012), Rahman et al. (2014) and Razzak et al. (2015) [36, 52–54] (Table 3), and the negative controls of meat products (chicken and beef meatball((≥ 50 g/piece), burger(≥ 100 g/piece) and frankfurter(≥ 80 g/piece)) were prepared in different three days using pure ground chicken and beef meat(blended by Pensonic Super Blender- PB-3205, 13600 prai, Penang Malaysia) along with a rational amount of fats and other culinary ingredients. Similarly, the positive controls were made by spiking 10%, 1%, 0.1%, and 0.01% of MBT meat into the chicken and beef meat, which was used to make various dummy meat products (Table 3). As presented in Table 3, culinary salt, garlic and other ingredients were added to the mixtures and blended vigorously until a homogenous mush was obtained, and the emulsified homogenous mixtures were then shaped into meatball, burger, and frankfurter products [54]. All samples were prepared in triplicate on three different dates by three independent analysts. All of the prepared meat products were subjected to autoclaving at 120°C under a pressure of 45 psi for 2.5 h, after which, all of the meatballs were stored at −20°C for further DNA extraction.

**Table 2:** Analysis of dummy and commercial meat products using MBT-specific PCR assay.

Items	Number of samples	Malayan box turtle DNA detection	Detection accuracy (%)
Meat products			
Pure chicken meatball	9	0/9	100
Pure beef meatball	9	0/9	100
MBT-spiked chicken meatball	27	27/27	100
MBT-spiked beef meatball	27	27//27	100
Pure chicken burger	9	0/9	100
Pure beef burger	9	0/9	100
MBT-spiked chicken burger	27	27/27	100
MBT-spiked beef burger	27	27/27	100
Pure chicken frankfurter	9	0/9	100
Pure beef frankfurter	9	0/9	100
MBT-spiked chicken frankfurter	27	27/27	100
MBT-spiked beef frankfurter	27	27/27	100
Commercial chicken meatball			
A	9	0/9	100
B	9	0/9	100
C	9	0/9	100
D	9	0/9	100
Commercial beef meatball			
E	9	0/9	100
F	9	0/9	100
G	9	0/9	100
H	9	0/9	100
Commercial chicken burger			
I	9	0/9	100
J	9	0/9	100
K	9	0/9	100
L	9	0/9	100
Commercial beef burger			
M	9	0/9	100
N	9	0/9	100
O	9	0/9	100
Commercial chicken frankfurter			
P	9	0/9	100
Q	9	0/9	100
R	9	0/9	100
Commercial beef frankfurter			
S	9	0/9	100
T	9	0/9	100
U	9	0/9	100

**Table 3:** Formulation of ready-to-eat model meat products.

Ingredients	Meatball (≥ 50 g/piece)		Burger (≥ 100 g/piece)		Frankfurter (≥80 g/piece)	
	Beef	Chicken	Beef	Chicken	Beef	Chicken
Minced meat	33 g^a^	33 g^a^	70 g^a^	70 g^a^	55 g^a^	55 g^a^
Soy protein	5 g	5 g	10 g	10. g	10 g	10 g
Starch/breadcrumb	6 g	6 g	8 g	8 g	7 g	7 g
Chopped onion^b^	2 g	2 g	4 g	4 g	2 g	2 g
Chopped ginger^b^	0.2 g	0.2 g	0.4 g	0.4 g	0.2 g	0.2 g
Cumin powder^b^	1 g	1 g	1 g	1 g	1 g	1 g
Garlic powder^b^	0.5 g	0.5 g	1 g	1 g	0.5 g	0.5 g
Black pepper^b^	0.15 g	0.15 g	0.3 g	0.3 g	0.2 g	0.2 g
Tomato paste	1.5 g	1.5 g	2.5 g	2.5 g	2 g	2 g
Butter	1.5 g	1.5 g	2.5 g	2.5 g	2.5 g	2.5 g
Egg			1 g	1 g		
Salt	SA	SA	SA	SA	SA	SA
Others^c^	SA	SA	SA	SA	SA	SA

^a^ 10%, 1%, 0.1% and 0.01% of MBT meat was added to a balanced amount of minced chicken and beef to make ≥ 50-g, 100-g, and 80-g specimens of each meatball, burger, and frankfurter, respectively.

^b^ Amounts are approximate, and some items were measured in teaspoons.

^c^ Enhancers and flavoring agents. SA-Suitable amount.

### Target stability test

All of the meat samples were individually cut into smaller pieces, and approximately 5–6 g of each sample were cooked at 100°C for 60, 90, 120 and 150 min to simulate traditional cooking. Secondly, all meat samples were extremely autoclaved at 120 °C under 45 psi for 2.5 h. Finally, microwave cooking was performed at 600, 650 and 700 W for 30 min, and all of the heat-treated samples were stored at -20°C until further use. Total DNA was extracted from 30 mg of the heat-treated samples using a Yeastern Genomic DNA Mini Kit (Yeastern Biotech Co., Ltd. Taipei, Taiwan). For medicinal products, 30 mg powder was used for DNA extraction. Finally, the concentration and purity of the extracted DNA samples were tested by UV-VIS spectrophotometer (Biochrom Libra S70, Biochrom Ltd, Cambridge, UK) based on an absorbance value of 1.7–2.0 at A260/280 nm and calculating the ratios. All the purified DNA was kept at -20°C until further use.

### DNA extraction and PCR optimization

Total DNA was extracted from 30 mg of muscle tissue of each species (21 species) and the admixed and commercial meat products using a Yeastern Genomic DNA Mini Kit (Yeastern Biotech Co., Ltd. Taipei, Taiwan), and plant DNA was extracted following Ma et al. (2000). The purity and concentration of all of the extracted DNA was determined using a UV-VIS spectrophotometer (Biochrom Libra S70, Biochrom Ltd, Cambridge, UK) based on absorbance at A260/280 and calculating the ratios [26]. All of the extracted DNA was kept at -20°C until further use. The PCR assay was optimized and performed in a 20-μl reaction mixture containing 1x PCR master mix (Promega, Corporation, Madison, USA) composed of 5 units μl^-1^ of Taq DNA polymerase (supplied in a proprietary reaction buffer of pH 8 containing 150 μM dATP, dGTP, dCTP, and dTTP and 1.25 mM MgCl_2_; 150 nM of each primer and 20 ng of extracted DNA). Additionally, 150 nM of each eukaryotic 18S rRNA primer was added into the reaction mixture as an endogenous internal control [51], and a negative template control was made using nuclease-free water in place of template DNA. The internal positive and negative controls were used to ensure the presence of good-quality DNA in each PCR tube to avoid contamination and detection of false negatives. The PCR assay was optimized in a Veritii 96-Well Thermal Cycler (Applied Biosystems Inc., Foster, CA, USA) with initial denaturation at 95°C for 3 min followed by 35 cycles of denaturation at 95°C for 20 s, annealing at 58°C for 20 s, extension at 72°C for 30 s and a final extension at 72°C for 5 min. Finally, the PCR products were visually detected in a gel doc instrument (AlphaImager HP, Santa Clara, CA, USA).

### Specificity and sensitivity tests of pure and admixed samples

The specificity of the designed primers was confirmed by basic local alignment search tool (BLAST) analysis, a multiple sequence alignment program and an optimized PCR assay, wherein the MBT primers were challenged against the DNA of 20 different non-target species (pond slider turtle, chicken, cow, goat, pig, pigeon, monkey, rat, cat, dog, sheep, duck, buffalo, deer, giant river prawn, cod, salmon, carp, wheat, and cucumber). Pairwise distances were calculated using the 120-bp site of the MBT cytochrome b gene against 28 different species (S1 Table). Additionally, a phylogenetic tree was constructed to study the maximum and minimum genetic distances between the MBT and 66 reptile species, of which 44 were turtles (1–44), 10 were snakes (48–57), eight were crocodiles (58–65) and five were lizards (45–47, 66–67)(S1 Fig). The sensitivity of the assay was determined using serially diluted DNA extracted from pure meat tissues (10 ng, 1 ng, 0.1 ng, 0.01 ng, 0.001 ng, 0.0001 ng) and the binary mixture, ternary mixture and commercial meat products (chicken and beef meatballs, burgers and frankfurters). All of the admixed meat samples were made by spiking the beef, chicken and goat meat with 10%, 1%, 0.1%, and 0.01% MBT meat (Table 1).

### Enzymatic digestion and RFLP analysis

The MBT-specific PCR product (120 bp) was digested with *Bfa1*-restriction endonucleases (New England Biolabs, Ipswich, MA, USA) in a 30-μl reaction mixture containing 12 μl of PCR product, 1 μl of restriction enzyme (1 FDU), 2 μl of 10x digestion buffer supplied with enzyme and 15 μl of distilled water. All of the mixtures were mixed properly and kept in a water bath at 37°C for 30 min, and DNA digestion was stopped by placing the mixture in another water bath at 80°C for 20 min. For the RFLP analysis, 1 μl of digested PCR product was applied to a microfluidic lab-on-chip well using a 1 K DNA analysis kit, and the desired fragments were separated in a Bio-Rad Automated Electrophoresis station (Experion, Bio-Rad, Inc., USA).

### Compliance with Ethics Requirements

Ethical clearance (ref. no: NANOCAT/23/07/2013/A(R)) was obtained from the Institutional Animal Care and Use Committee, University of Malaya (UM IACUC), and all experiments were conducted following the national and institutional guidelines for handling animal meats used in this study.

## Results and Discussion

### DNA extraction

Total genomic DNA was extracted from pure and admixed (binary, ternary and MBT-mixed) meat products under raw and processed (boiled, autoclaved and microwaved) states. The specimens were prepared on three different dates by three independent analysts as documented in our earlier report [26]. The concentration and purity of the extracted DNA were determined, and the 260/280-nm absorbance of all of the samples was 1.7–2.0 which indicates good quality DNA [55]. DNA yield was higher from the heat-treated meat samples (220–350 ng/μl) than from the raw meat samples (150–210 ng/μl) probably due to sample dehydration, which increases the effective number of cells and, thus, the number of analytes per unit weight of the treated samples [56].

In contrast to the meat and meat products, the concentration of the extracted DNA from medicinal samples was 10–35 ng/μl and the purity of the genomic DNA was 1.70–1.75 (data not shown); this might be referred to the multiple components such as polysaccharides that may not contain DNA. Furthermore, the various plant and animal materials might involve inhibitors and also the series of processing procedures such as drying and stewing that definitely degrade DNA to variable extents. We also encountered difficulties in dissolving 14 jelly powder samples which frequently precipitated during DNA extraction process. This was not a surprise since TCM preparations often involve decoction method that extensively modifies the natural composition and textures of the source ingredients, bringing in many excipient species. Therefore, the DNA extraction method was modified by increasing lysis time in GT buffer and prolonging binding time to the nitrocellulose membrane in GD column using GBT buffer (Yeastern Biotech Co., Ltd. Taipei, Taiwan); using this modified protocol, 36 of 50 jelly powder samples were dissolved and DNA extraction was successful. However, the concentration of the DNA from these jelly powder samples was 10–15 ng/μl, which was far less than that from other medicinal samples that yielded 20–35 ng/μl.

### Malayan box turtle-specific PCR assay

**Fig 2:**
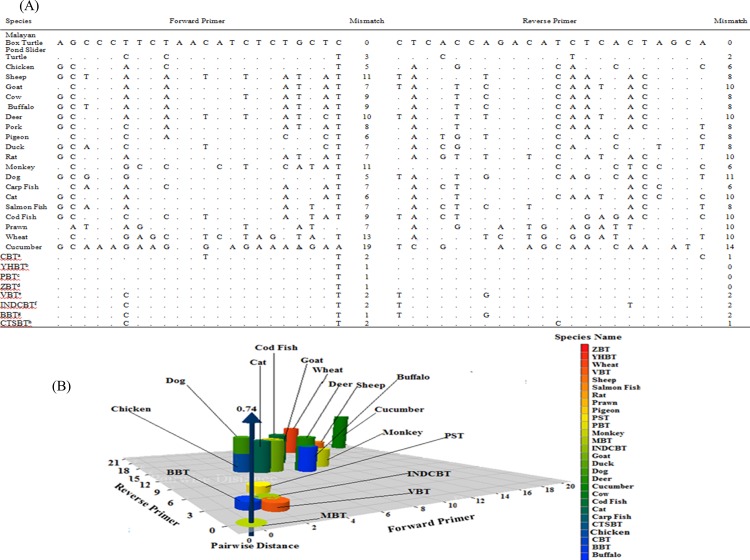
**(A) The mismatch comparison of the Malayan box turtle-specific forward and reverse primers tested against 20 non-target and 8 *Cuora* species. (B) 3D plot showing the primer mismatch and pairwise distance.** In (A), ^*a*^*Cuora flavomarginata*-Chinese box turtle (CBT), ^b^*Cuora aurocapitata*-yellow-headed box turtle (YHBT), ^c^*Cuora pani-*Pan’s box turtle (PBT), ^d^*Cuora zhoui*-Zhou's box turtle (ZBT), ^e^*Cuora picturata*-Vietnamese box turtle (VBT), ^f^*Cuora galbinifrons*-Indochinese box turtle (INDCBT), ^g^*Cuora bourreti*-Bourret’s box turtle (BBT) and ^h^*Cuora trifasciata*-Chinese three-striped box turtle(CTSBT) are shown. In (B), 3D plot showing the primer mismatch and pairwise distance between the Malayan box turtle and 20 studied species including 8 species of the *Cuora* genus.

The species-specific PCR assay is a simple and low-cost technique that could be performed in most laboratories; it is often conclusive and has been widely used for meat speciation. Currently, simplex [26] and multiplex PCR assays [36, 57] have been proposed for the authentication of common meats in the food chain, and in the species-specific PCR systems, the development of effective primers is often key[58]. Studies demonstrate that even a single base mismatch at the 3′ end can compromise PCR efficiency and/or often results in amplification failure [59], so considering this pitfall, this report critically evaluated the mismatched bases in the primer annealing regions (Fig 2A). The designed primer pairs were aligned against 28 potential non-target species, including 8 closely related species of the *Cuora* genus, using the ClustalW multiple alignment program; it is noteworthy that all 12 of the species of the *Cuora* genus are critically endangered [14]. The available genetic sequences of 9 *Cuora* species in NCBI were subjected to mismatch analysis (Fig 2A) for the study of pairwise distance (S1 Table), the phylogenetic tree (S1 Fig) and the 3D plot (Fig 2B), which reflected very close matching among the 9 *Cuora* species. Thus, there is a high chance of detecting all of the *Cuora* species with the MBT-specific primers, so there was a need for experimental probing by cross-testing all of the *Cuora* species. However, due to the unavailability of samples and lack of permission, this practical evaluation could not be executed. Close observation of the sequences indicated 1–4 nucleotide (nts) mismatches among the *Cuora* species, and this low number of mismatched nts in the primer annealing region was probably due to some trait modification due to habitat loss and consequent adaptation to a new environment, gene translocations and wildlife farming, which leads to increased rates of anthropogenic hybridization and introgression among native and introduced animals within the same genus [14]. Although even a single-nt mismatch at the 3′ end of a primer may prevent successful PCR amplification [59], most of the mismatches (1–4 nt) among the *Cuora* species were at the middle position of the reverse primer (Fig 2A) and thus had little effect on PCR amplification. This was evidence that all of the *Cuora* species might be detected by the MBT-specific primers, but this did not compromise the assay of interest. Rather, it increased the scope of the assay many fold because all of the species of the *Cuora* genus are critically endangered, so finding a universal PCR assay for their detection is extremely desirable. A phylogenetic tree that was constructed using similar sites of the cytb genes of 66 reptile species, of which 44 were tortoises and turtles, 10 were snakes, 8 were crocodiles and 5 were lizards, clearly showed the genetic relationship between MBT and the other reptile species (S1 Fig). The constructed genetic map demonstrated a close relationship among the 9 *Cuora* species and a huge distance between the MBT and the other reptile species. This reflected a strong probability of *Cuora* genus identification without cross-amplifying any of the reptiles or other species.

**Fig 3:**
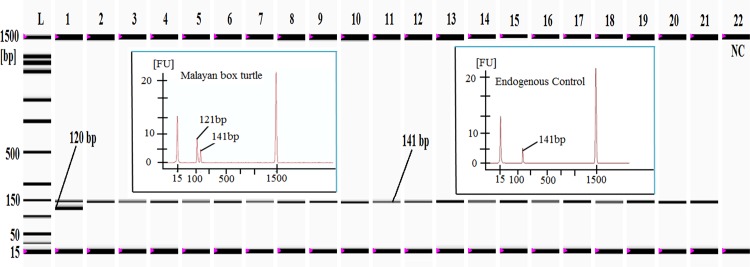
Cross-specificity analysis of Malayan box turtle (MBT)-specific primers against 20 different non-target animal and plant species. *Lane L*: ladder DNA (15–1500 bp) and *lanes 1–21*: PCR products from the MBT target (120 bp) and eukaryotic endogenous control (141 bp). Please note that the Malayan box turtle-specific product was only amplified from the Malayan box turtle (*lane* 1), but the endogenous control was obtained from the Malayan box turtle, the pond slider turtle, chicken, lamb, goat, cow, buffalo, deer, pig, duck, pigeon, dog, monkey, cat, rat, salmon, carp, cod, prawn, wheat and cucumber (*lanes 1–21*, respectively). *Lane 22*: negative control (NC).

Among the 21 tested species, 100% sequence matching was only obtained with the cytb gene of the Malayan box turtle, and multiple mismatches (5–33 nt) were found with the non-target species (Fig 2A). The BLAST study against the non-redundant nucleotide sequences (data not shown) in NCBI reflected a similar result. The ClustalW and BLAST analysis results were experimentally authenticated by a practical PCR experiment using 20-ng template DNA of the target and non-target species extracted from raw and various types of processed meat. In repeated experiments, a 120-bp PCR product was only obtained from Malayan box turtle; other species did not yield this specific product (Fig 3). Amplifiable DNA in non-target species was confirmed from an endogenous control that amplified 141-bp PCR products from all species using a set of universal eukaryotic primers of the 18S rRNA gene [51].

The sequence, pairwise distance and 3D plot (Fig 2B) of the MBT 120-bp PCR product [60, 61] showed minimum distances between the Malayan box turtle (MBT) and the Chinese box turtle (CBT), the yellow-headed box turtle (YHBT), Pan’s box turtle (PBT), Zhou's box turtle (ZBT), the Vietnamese box turtle (VBT), the Indochinese box turtle (IBT), Bourret’s box turtle (BBT), the Chinese three-striped box turtle (CTSBT), chicken, buffalo and the pond slider turtle (PST) (0.06–0.20); a maximum distance was found between MBT and cucumber (0.74). These data clearly reflected a large genetic distance, demonstrating the unlikelihood of cross-species amplification in a practical PCR assay. The number of mismatched bases in the primer-binding sites of the studied species was between 11.4–75% or 5–33 nucleotides, which made the likelihood of cross-species amplification improbable (Fig 2A). The phylogenetic tree constructed by the neighbor-joining method (not shown) also demonstrated a high degree of discrimination among MBT and the other animal, fish and plant species.

Finally, the theoretical results were experimentally validated by an authentic PCR test against the target and 20 different non-target species using 20 ng of DNA extracted from all of the tested samples. A 120-bp PCR product was only found from the MBT sample, and such a product was absent in all of the other species (Fig 3). Meat products could contain various types of chemical compounds such as the Maillard reaction product, milk proteins, glycogen, fat, collagen, fulvic acids, and iron, which may be co-purified with the target DNA and inhibit the PCR [62]. However, the use of both the negative and positive controls in all of the PCR assays eliminated any possibility of a false positive or negative target detection. The presence of amplifiable DNA in non-target species was confirmed from an endogenous control that amplified the 141-bp PCR product from all of the species through a set of universal eukaryotic primers of the 18Sr RNA gene. Several methods such as PCR[20] (12S rRNA, 165 bp and cytb, 376 bp), PCR-RFLP [49] (cytb, 876 bp) and PCR product sequencing [63] (cytb, 405 bp) have been successful for the detection of turtle and tortoise species including the Malayan box turtle. However, longer targets (≥ 165 bp), which are frequently fragmented during processing treatments, have been used in earlier reports [26], and such documented assays are not suitable for the authentication of meat species under food-processing conditions [64]. Furthermore, the sensitivity and stability of those assays have not been clearly described. Here we defined the experimental detection limit and the stability of the MBT-specific PCR assays in binary and ternary admixtures as well as traditionally consumed beef and chicken meatball, burger and frankfurter products under raw and heat-processed conditions. We only obtained the MBT-specific PCR product (120 bp) from samples that were deliberately made with MBT meat.

### Limit of detection

**Fig 4:**
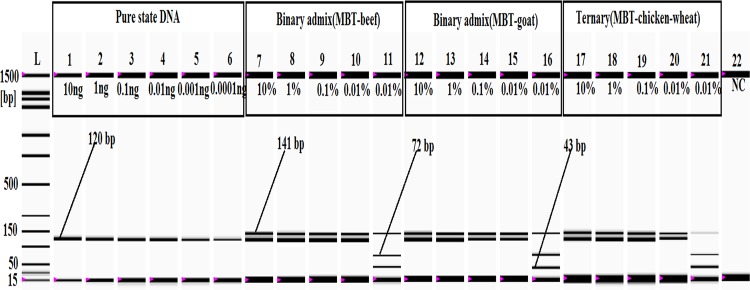
Sensitivity analysis of pure, binary and ternary admixtures. In the gel image, *lanes 1–6*: PCR products from 10, 1, 0.1, 0.01, 0.001, and 0.0001 ng MBT DNA, respectively. *Lanes 7–10* (MBT and beef) and *lanes 12–15* (MBT and goat) represent PCR products from 10%, 1%, 0.1%, and 0.01% MBT-adulterated binary admixtures, respectively. The *Bfa1* digestions of the MBT-specific PCR product realized from 0.01% MBT admixed with beef and goat are shown in *lanes 11 and 16*, respectively. In the gel image, *lanes 17–20* represent PCR products from the ternary mixture (MBT, chicken and wheat flour) containing 10%, 1%, 0.1%, and 0.01% MBT meat, and *lane 21* shows the *Bfa1* digestion of the MBT-specific PCR products obtained from the ternary admix containing 0.01% MBT. *Lane L*: ladder DNA, and *lane 22*: negative control (NC).

The limit of detection (LOD) of an assay is a critical aspect of the determination of marginal-level targets in adulterated foodstuffs, and the LOD values for several types of animal species, such as beef[65], chicken, turkey [66, 67], goat[68], lamb and pork [69], deer[51] and wild boar [70], have been defined for food authenticity studies. However, the Malayan box turtle is a relatively new species in food chains, so its LOD has not been defined under various food matrices. All of the previous assays for the detection of MBT and other turtle species have described the evolutionary origins [14], taxonomy [15] and phylogenetic [20] relationships among the closely related species, so this study addressed this research gap by determining the LOD in two different ways. Firstly, the concentration of the extracted DNA was measured by UV-VIS spectrophotometer at a relatively high concentration (100 ngμl^-1^) (Biochrom Libra S70, Biochrom Ltd, Cambridge, UK), and then various concentrations (10, 1, 0.1, 0.01, 0.001, 0.0001 ng) were made by dilution in nuclease-free water because inaccuracies and inconsistencies have been observed in spectrophotometric readings using low concentrations. A 10-fold serial dilution method has been used by several studies to determine the PCR sensitivity for porcine, mutton [69], monkey [39] and cat species [71], and in this study, the amplified PCR product was found from an amount as low as 0.0001 ng DNA extracted from pure meat (Fig 4). Previously, Ali et al. (2012) and Raifana et al. (2015) detected 0.0001 ng of porcine and monkey DNA in pure meat [39, 52]. The sensitivity of this newly designed PCR assay for MBT detection indicated that the actual LOD was higher than those in published reports [26].

The aim of the study was to detect a minimal amount of adulterated MBT in raw and processed meat products, so three sets of base adulterated meat mixture (BAM) were made following Ali et al. (2012 and 2015a,b) to simulate the most likely forms of adulteration. Set (1) was a turtle-beef binary admixture; set (2) was a turtle-goat binary admixture, and set (3) was a turtle-chicken-wheat ternary admixture [26, 36, 52]. Fig 4 shows the MBT-specific PCR products from the binary (MBT-beef and MBT-goat) and ternary admixtures (turtle-chicken-wheat flour), and all of these results clearly supported the high sensitivity and specificity of the MBT-specific primers developed in this study because they amplified the specific target product from admixtures containing MBT meat in concentrations as low as 0.01% (w/w) under complex matrices. From a practical point of view and also based on published reports, it is clear that meat products that are adulterated by less than 0.1% do not yield remarkable profits in the food industry, and it is very difficult to prepare admixed samples with less than 0.1% contamination. However, the findings obviously suggested that this assay could be used to detect adulterations much lower than 0.1% (w/w). Previously, Rashid et al. (2015) detected 0.1% (w/w) monkey DNA under various admixed states, and Ali et al. (2012) identified up to 0.01% (w/w) pork under different food matrices[39, 52]. In contrast, Karabasanavar et al. (2011) obtained PCR product from 0.1% (w/w) mutton mixed with cattle, buffalo, goat pig, and chicken [68], and Mane et al. (2012) found less than 1% beef adulteration in admixed meat and meat products [65]. Thus, the LOD established in this study was far below those from the published reports. Furthermore, to verify the specificity, the PCR products from the 0.01% (w/w)-admixed samples were restriction digested with *Bfa1*-restriction endonuclease, and distinctive DNA fragments or identifiable MBT fingerprints were obtained (Fig 4).

### Evaluation of meatballs, burgers and frankfurters

**Fig 5:**
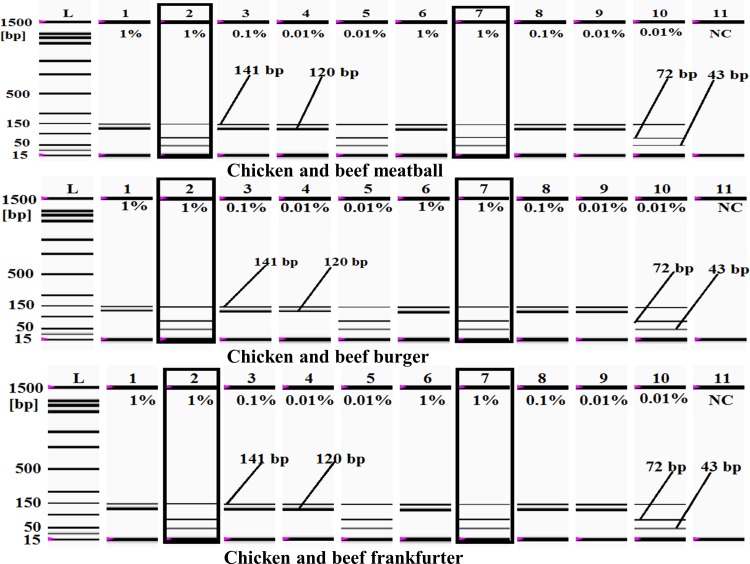
**a, b and c. MBT meat analysis in model meat products**. In all figures (a-c), *lanes 1*, *3*, *4* and *6*, *8*, *9* are PCR products from 1%, 0.1% and 0.01% MBT meat-spiked chicken (*lanes 1*, *3* and *4*) and beef (*lanes 6*, *8*, *9*) meatballs (a), burgers (b), and frankfurters (c), respectively. *Lanes 5* and *10* represent the *Bfa1* digestion of the PCR products obtained from 0.01% MBT meat-spiked chicken and beef meatballs (a), burgers (b), and frankfurters (c). *Lanes 2* and *7* are the *Bfa1* digestion of the PCR products obtained from 1% MBT meat-spiked chicken and beef meatballs (a), burgers (b), and frankfurters (c) after autoclaving. *Lane L*: ladder DNA, and *lane 11*: negative control (NC).

To establish the validity, stability and reliability of the developed PCR-RFLP assay, various commercially available meat products including beef and chicken meatballs, burgers and frankfurters, which are widely consumed across Malaysia, Indonesia, China and most of the world, were experimentally screened [33, 53, 54, 72]. Eight (8) commercially available halal-branded chicken and beef meatballs (A-H), seven (7) chicken and beef burgers (I-O) and eight (6) chicken and beef frankfurters (M-T) were collected from four different Malaysian outlets located in the states of Kuala Lumpur and Selangor in Malaysia on three different days (Table 1). The adulteration of meat products was simulated in dummy commercial meat products following Ali et al. (2012), Rahman et al. (2014), and Razzak et al. (2015), in which dummy meatball, burger and frankfurter products were spiked with 10%, 1%, 0.1% and 0.01% of ground MBT meat [52–54]. Additionally, the 1% MBT-spiked meat products were subjected to autoclaving for 2.5 h at 120°C under 45 psi (Fig 5A, 5B and 5C) because this treatment is known to break down DNA [26, 64]. The MBT-specific PCR product was amplified from all levels of adulteration including the (1%) autoclaved samples. However, no MBT-specific PCR product was observed from the meat products collected from commercial sources (Table 1). To confirm the origin of the amplified target, the PCR products were digested with *Bfa1*-restriction endonuclease enzyme, and distinctive MBT fingerprints composed of 72, 43 and 5-bp oligo fragments were obtained from all of the meat products, but only the 72- and 43-bp products were detected in the gel image due to the inability of the currently available analytical machine to detect a 5-bp product (Fig 4, Fig 5A, 5B and 5C) [39]. These digestion results completely matched the *in silico* digested fragments (not shown), so the findings strongly supported that the PCR-RFLP assay was specific to MBT and suitable for identifying less than 0.01% (w/w) MBT meat adulteration in commercial food. The endogenous control specific to eukaryotic 18S rRNA does not contain the *Bfa1* cutting site, so a clear 141-bp product was detected in all samples [26]. All of the experiments were carried out in triplicate by three independent analysts on three different dates to confirm the reproducibility of the results [52]. The experimental and theoretical specificity, stability and sensitivity of the developed assay indicated that it was a reliable and rapid technique for the authentication of MBT adulteration in the food chain. Malaysia is committed to developing a halal hub industry and to being a competitive partner in the global halal food business, so the absence of MBT meat in Malaysian food was quite encouraging.

### Stability analysis

Target stability is a key point to consider in the validation of any analytical results, especially for forensic samples where less stable analytes are frequently decomposed resulting in false negative identifications. Published reports reflect the significant effort that scientists have put into the development of short-length DNA targets, which are thermodynamically more stable than the longer targets and hence persist through extreme stresses that often break down longer DNA markers [26]. In several instances, DNA barcoding has failed to recover longer targets from degraded specimens because of the longer and fragile attributes of the targets, which are often more than 600 bp in length[73]. Furthermore, the interference of nuclear integrated mitochondrial pseudogenes (numt) seriously compromises the reliability of DNA barcoding in species authentication [74], so instead of targeting universal markers, species-specific short amplicon-length PCR assays [36] and short-length barcode markers [75] have been prioritized over the years. In food forensic analysis, various thermally treated samples have been used to benchmark target biomarker stability [64, 76, 77], but because there are very few PCR assays available for the analysis of MBT in the food chain, there is a gap in the evaluation of the stability and robustness of the MBT-specific targets under various food processing conditions. This study confirmed the stability of the MBT target through three different thermal treatment schemes, namely, boiling, microwave cooking and autoclaving. Boiling simulates traditional cooking, in which meat is cooked in boiling water at 100°C for a fixed amount of time [26], and over the years, steam cooking or boiling have increased in popularity over pan frying for health reasons. In contrast, microwave cooking is a modern technique that heats and cooks food through exposure to electromagnetic radiation in the microwave spectrum[26, 39], while autoclaving is the most appropriate method to simulate steaming and canning-based meat processing because it cooks at a high temperature (121°C) under pressurized conditions to kill any potential microbes in the samples. Extreme autoclaving (2.5 h at 120°C and 45 psi) has been used as a benchmark for target DNA stability in several studies [36, 52, 71, 78].

**Fig 6:**
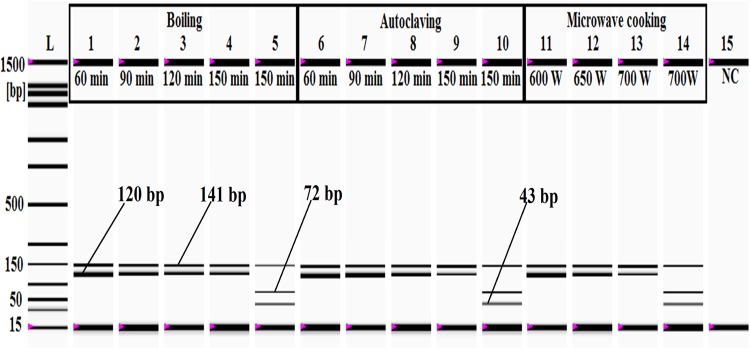
Stability analysis of the MBT-specific target DNA (120 bp) under boiling, autoclaving and microwave cooking. In the gel image, *lanes 1–4* are boiling at 100°C for 60, 90, 120 and 150 min, respectively, and *lanes 6–9* are autoclaving at 121°C for 60, 90, 120 and 150 min under a pressure of 45 psi. *Lanes 5* and *10* represent *Bfa1* digestion from samples boiled (*lane 5*) and autoclaved (*lane 10*) for 150 min, respectively. In the gel image, *lanes 11–13* represent microwave cooking at 600 W, 650 W and 700 W for 30 min, respectively, and *lane 14* is the *Bfa1* digestion of the microwave cooked sample at 700 W. *Lane L*: ladder DNA, and *lane 15*: negative control(NC).

In this study, the MBT-specific target was obtained from all of the thermally processed samples (Fig 6); when MBT meat was boiled at 100°C for 60, 90, 120 and 150 min, no adverse effects on the amplification cycle were found (Fig 6). Previously, Ali et al. (2012) detected a 109-bp porcine target after boiling for 2.5 h, and Haunshi et al. (2009), Karabasanavar et al. (2011) and Mane et al. (2012) identified target species after autoclaving various types of domestic meat at 121°C for 15–30 min [52, 68, 76]. Here, we autoclaved MBT meat at 120°C for 60, 90, 120 and 150 min (extensive treatment) under 45 psi and obtained targeted PCR products from all of the treated samples (Fig 6). Finally, extreme microwave cooking was done at 600–700 W for 30 min, and clear a band for the desired product (120 bp) was obtained (Fig 6). In an earlier report, we showed that short-length targets are more stable than longer ones [26], but when meat samples were cooked in a microwave at 700 W for 30 min, they turned into ashes that were no longer suitable for consumption (data not shown). Previously, Arslan et al. (2006) failed to amplify the target product from pan-fried beef meat at 190°C for 80 min [64], but in this assay, target amplification from ash-like specimens was a clear indication that this method could be used to detect the MBT target from highly decomposed specimens, which are frequently found in forensic samples.

### Product authentication by PCR-RFLP assay

Species-specific PCR assay is often conclusive [26], but it has yet to be considered a definitive analytical method due to certain “hard-to-control” features of the amplification process [26, 78]. For example, it sometimes produces artifacts due to contamination by alien DNA at a minute scale [78, 79], but these ambiguities or doubts could be eliminated by the verification of the amplified product through at least one of three different methods, namely, PCR-RFLP assay, probe hybridization, and target product sequencing [80]. Probe hybridization is an attractive technique because it can detect multiple species in a single experimental run through the use of multiple labeled probes [81], but this procedure requires purified DNA and is also laborious, expensive and time-consuming [39]. In contrast, DNA sequencing is a more efficient and reliable tool, but it requires an expensive laboratory set-up and is often not suitable for the analysis of processed food under complex matrices [82, 83] because co-extracted food ingredients can complicate the results [84]. In contrast, the PCR-RFLP assay can overcome all of these limitations and has been widely used to authenticate the original PCR product amplified from a particular target [85–87]. It comprises the generations of a specific fragment profile through restriction digestion with one or two endonucleases. A carefully selected restriction endonuclease cleaves the PCR product at specific recognition sites, producing a set of DNA fragments of different lengths that could be separated and visualized by gel electrophoresis [88], so it distinguishes the artificial PCR product from the original through the analysis of the restriction fingerprints[32, 89]

In this study, we digested the 120-bp MBT-specific PCR product by the *Bfa1*-restriction endonuclease enzyme (New England Biolabs, see http://nc2.neb.com/NEBcutter2/) because *in silico* analysis showed two restriction sites for the *Bfa1* enzyme with unique fragment lengths: 72 bp, 43 bp and 5 bp. The digested products (72 bp, 43 bp and 5 bp) obtained from the MBT-specific PCR products (120 bp) were separated and visualized by a micro-fluidic chip-based automated electrophoresis station (Bio-Rad Laboratories, Inc., USA) (Figs 4–6), but because the 5-bp fragment size was below the resolution capacity of the instrument (bioanalyzer), it remained undetected in the gel image (Figs 4–6). The endogenous target (141-bp) was amplified from all of the analyzed samples, which indicated the presence of good quality DNA and thus ruled out the probability of any false negative results. Two sets of binary mixed-meat products (MBT-beef and MBT-goat) and 1 set of a ternary mixed-meat product (MBT-chicken-wheat) were made to emulate the most likely forms of adulteration in processed foods. In the admixtures, 10%, 1%, 0.1% and 0.01% of the MBT meats were spiked in a balanced amount of deboned beef and goat, whereas 10%, 1%, 0.1% and 0.01% of MBT meat were added to chicken and wheat flour at ratios of 20:80:100, 2:98:100, 0.2:99.8:100 and 0.02:99.98:100 (Fig 4). The MBT-specific PCR product (120 bp) was obtained from all forms of adulteration at a tested limit of detection of 0.01% MBT meat; all of the PCR products were validated by digestion with the *Bfa1* enzyme (Fig 4). We further screened for MBT DNA in various types of food products using laboratory prepared dummy meat products and commercially available meat products (Tables 1 and 2). Lower-value meats are commonly used to replace higher-value meats to increase economic profit [52], so the most popular meat products, such as chicken and beef meatballs, burgers and frankfurters, were prepared following Ali et al. (2012), Rahman et al. (2014) and Razzak et al. (2015) [52–54]. All of these meat products were spiked with 10%, 1%, 0.1% and 0.01% MBT meat to demonstrate the common forms of adulteration in the commercial food industry. However, mixing with less than 1% of low-value meat does not yield any significant economic benefit relative to the great risk of defamation[54]. Consequently, we determined that the sensitivity of this assay using 1% MBT-adulterated autoclaved dummy meats was suitable for the detection of trace level adulteration in processed food. The RFLP results indicated clearly unique fragment patterns specific to MBT in 1% MBT-adulterated meat products (Fig 5).

### Traditional Chinese medicines analysis

**Fig 7:**
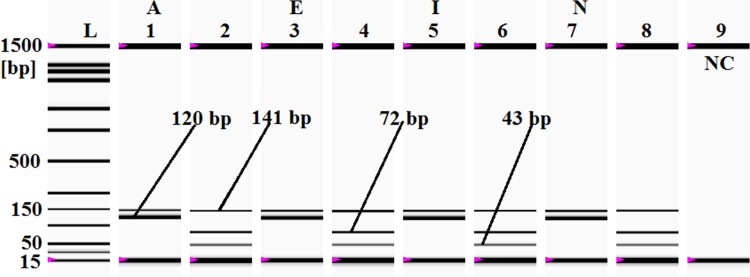
Traditional Chinese medicines analysis. In the gel image, *lanes 1*, *3*,*5 and 7*are *for A*(Chinese herbal jelly) *E* (anti inflammation) *I* (Sex stimulator) *& N (*Leukorrhea) medicines respectively, and *lanes 2*,*4* and *6* represent *Bfa1* digestion from samples *A E I* & *N* respectively. *Lane L*: ladder DNA, and *lane 9*: negative control (NC).

The wide spread trades of the various traditional Chinese medicines (TCMs), which are claimed to provide multicure, has become a disastrous threats to the existence of several wild species, including MBT. Although most of them are claimed to be plant products, several highly endangered species, such as rhinos, crocodiles, turtles, tigers and elephants which are enlisted in the CITES Appendix I and II, have continued to be killed to supply the raw materials of these medicines which has got huge market in the Southeast Asia and Chinese communities around the world [90]. In many cases, enforcement of regulation has become impossible because the endangered animals are sold in forms, such as powder and jellies made from ground bones, shells and skins, which are difficult to be detected by customs officials [90–91]. Therefore, we attempted to screen MBT materials in 153 traditional Chinese medicinal products of 17 different brands sold in various Chinese medicines shops across Malaysia (Table 4). Sixty two (40%) of the 153 tested products were found to be MBT positive, reflecting the wide spread consumption and uses of MBT materials in these medicines, but the information was hidden in the product labels (Table 4). To verify the authenticity, the amplified PCR products of the MBT positive samples were digested with *Bfa1*-restriction endonuclease enzyme and distinctive MBT fingerprints (72 and 43 bp) were obtained (Fig 7) (Table 4). Since herbal products are regarded as low-risk and natural sources of cure for many diseases, traditional medicines are not under the stringent regulation. In this regard, the TCMs studied here clearly reflect that such declarations are not correct at all times; the graving concern is that MBT ingredients were not declared in the labels and the most of these preparations were claimed to be plant products to prove that there are no religious obligations since plant products are permitted in all religions. Recently, an Australian study found that high rates of adulteration (90%), substitution and mislabeling are rampant in TCMs, wherein the undeclared ingredients were either illegal or potentially hazardous to the consumers [92–93]. Thus the 62 TCM samples tested in our lab provided a 100% matching of DNA materials with *Cuora amboinensis* species, reflecting a clear break of wildlife conservation law in the preparation and selling of TCMs [94–96]. This is also contrary to the regulation of the US Food and Drug Administration (FDA) and European [97–99] laws, which require a mandatory declaration of the product ingredients. Report has been published that wild and domestic animals and their by-products such as hooves, skins, bones, feathers and tusks are used in the preparation of curative, protective and preventive medicines [100], causing over-hunting and massive threats to wildlife [97–99]. Moreover, some organs and animal by-products, such as bones and bile, can be a source of *Salmonella* infection that causes chronic diarrhoea and endotoxic shock. In this context, the possibility of transmitting infections or ailments from animal preparations should be seriously considered [101].

**Table 4:** Analysis of traditional Chinese medicines using MBT specific PCR assay.

Code of medicinal items	Information of the products labeling	Product applications	Number of samples	Malayan box turtle DNA detection
A	Chinese herbal jelly powder	Nocturnal enuresis, Anti-inflammation, Dessert soup, Muscle growth, Relieving itching, Reducing acne and kidney restoration, Blood circulation,	9	5/9
B	Chinese herbal jelly powder	Nocturnal enuresis, Anti-inflammation, Dessert soup, Muscle growth, Relieving itching, Reducing acne and kidney restoration, Blood circulation	9	4/9
C	Herbal jelly powder	Nocturnal enuresis, Muscle growth, Relieving itching, Reducing acne and kidney restoration, Blood circulation, Anti-inflammation	9	4/9
D	Herbal jelly powder	Nocturnal enuresis, Anti-inflammation, Dessert soup, Muscle growth, Relieving itching, Reducing acne and kidney restoration, Blood circulation, Anti-inflammation	9	3/9
E	Semen nulumbinis, Mel, Frutus Lycii, Radix ginseng, Cordyceps, Radix astragali, Radix codonopsis pilosulae, Radix morindae	Anti-inflammation, Pumpils, Blood Circulation, Male fertility, Strength of knees, Gall bladder, Hepatitis, Herpes, Shingles, Otitis meida, Cystitis, Hyperthyroidism, Migraines and Jaundice	9	3/9
F	Rhizomz dioscoreae, Fructus ziziphi jujubae, Radix ginseng, Cordyceps, Radix astragali, Radix codonopsis pilosulae, Radix morindae	Anti-inflammation, Pumpils, Gall bladder, Hepatitis, Herpes, Shingles, Otitis meida, Cystitis, Hyperthyroidism, Migraines and Jaundice	9	4/9
G	Semen nelumbinis, Radix ginseng, Cordyceps, Radix astragali, Radix codonopsis pilosulae, Radix morindae	Gall bladder, Hepatitis, Herpes, Shingles, Otitis meida, Cystitis, Hyperthyroidism, Migraines and Jaundice, Anti-inflammation, Pumpils	9	5/9
H	Radix ginseng, Cordyceps, Radix astragali, Radix codonopsis pilosulae, Radix morindae	Gall bladder, Hepatitis, Herpes, Shingles, Otitis meida, Cystitis, Hyperthyroidism, Migraines and Jaundice, Pumpils	9	0/9
I	Tongkat ali powder	Stimulate libido, Promote semen quality, Muscle growth, Pumpils, Blood circulation, Male enhancement	9	6/9
J	100% Tongkat ali capsule powder	Stimulate libido, Promote semen quality, Muscle growth, Anti-inflammation, Blood circulation, Male fertility	9	3/9
K	Tongkat ali capsule powder	Stimulate libido, Promote semen quality, Muscle growth, Anti-inflammation, Blood circulation, Male fertility	9	5/9
L	100% Tongkat ali capsule	Stimulate libido, Promote semen quality, and Muscle growth, Anti inflammation, Blood circulation, Male fertility	9	4/9
M	100% Tongkat ali powder	Stimulate libido, Promote semen quality, Muscle growth, Anti-inflammation, Blood circulation, Male enhancement and fertility	9	5/9
N	Radix gentinae, Radix bupleuri, Radix scutellariae, Fructus gardenia, Semen plantaginis, Radix angelicae sinensis, Radix rehmsnniae, Radix glycyrrhizae	Deafness, Hypochondriac pain, Irritability, Headache, Dizziness, Swollen sensation in ears/head, Swollen genitalia, Leucorrhea,	9	4/9
O	Radix gentinae, Radix bupleuri, Rhizomzz alismatis, Caulis clematidis, Semen plantaginis, Radix angelicae sinensis, Radix, Radix glycyrrhizae	Hypochondriac pain, Irritability, Headache, Dizziness, Deafness, Swollen sensation in ears/head, Swollen genitalia, Leucorrhea, Acute icteric hepatitis, Urethritis	9	0/9
P	Radix scutellariae, Fructus gardenia, Rhizomzz alismatis, Caulis clematidis, Semen plantaginis, Radix angelicae sinensis, Radix rehmsnniae, Radix glycyrrhizae	Liver, Gallbladder fire, Headache, Tinnitus, Hypochondriac pain, Irritability, Dizziness, Deafness, Swollen genitalia, leukorrhea	9	4/9
Q	Radix gentinae, Radix bupleuri, Radix scutellariae, Fructus gardenia, Rhizomzz alismatis, Caulis clematidis, Semen plantaginis, Radix angelicae sinensis, Radix rehmsnniae,	Migraine, Headaches, Acute pelvic inflammation, Swollen testes, Leukorrhea, Hyperthyroidism, Acute conjunctivitis, Acute liver fire	9	3/9

## Conclusions

The Malayan box turtle *(Cuora amboinensis)* is one of 18 native freshwater turtle and tortoise species in Malaysia, and despite being protected, it has been overexploited for its various organs, such shells and bones, which are believed to have healing properties for use in analgesic, antipyretic and invigorating traditional medicines. The highest rate of exploitation has been reported in East Asian countries for use in sex stimulants and invigorating traditional Chinese medicines, so the population of this species has significantly declined, putting it at risk of extinction. Moreover, its slow reproductive cycle due to late maturity and the production of few eggs has placed the species on the verge of disappearance, causing enormous potential harm to biodiversity. Therefore, it is feared that continuous, high-volume exploitation combined with its vulnerable life history characteristics might lead to a serious population decline, making the MBT extinct, at least locally.

The Malaysian government banned the hunting of this species for sale in domestic or international markets, but it clandestine trade under the labels of permissible meat is greatly suspected. Thus, there is a need for a reliable tracing method for MBT identification before this vulnerable species disappears. This study has developed a PCR-RFLP assay for the detection of MBT under complex food matrices, and the features inherent to the short DNA target (120 bp) of multicopy mitochondrial cytochrome b have made the target extraordinarily stable under various environmental and food processing stresses. Furthermore, the use of an endogenous control (141 bp) effectively eliminated the chances of a false negative detection, and false positive identification was similarly avoided through the use of a negative control. A phylogeny against 64 species, a mismatch analysis against 28 species and an experimental validation against 21 different types have made the assay highly reliable and viable even under severe conditions. Additionally, the lower limit of detection (0.1% MBT meat and 0.0001 ng DNA) has made the assay suitable for the detection of marginal levels of adulteration in popular food products such as meatballs, burgers and frankfurters. The study emulated the real forms of adulteration practices by making binary and ternary admixtures with various percentages of the target meat in a wide range of food products. Commercially available meat products and traditional Chinese medicine samples were screened to optimize and establish the validity of the assay for the analysis of marketed food, and the distinctive *Bfa1*-restriction profiles further authenticated the origin of the amplified products. Thus, it might be reasonable to claim that such a PCR-RFLP assay could be used by regulatory bodies, archaeologists and wildlife protection agencies for the detection of the Malayan box turtle under any matrices with great reliability and confidence.

## Supporting Information

S1 Fig Evolutionary distances between Malayan box turtle and other reptiles. **1–44: turtles/tortoises; 48–57: snakes; 58–65: crocodiles; and 45–47 and 66–67: lizards.** Phylogenetic tree was constructed using the Malayan box turtle-specific 120-bp site of the cytochrome b gene against 66 reptile species, of which 44 were turtle (1–44), 10 were snake (48–57), 8 were crocodile (58–65) and 5 were lizard species (45–47 and 66–67). Whereas a very narrow genetic gap was found among the 9 species of the *Cuora* genus, a wide genetic distance distinguished MBT from the other reptile species.
(PDF)

S1 Table Pairwise distances of the Malayan box turtle (MBT)-specific primer sites of the cytochrome b gene against the corresponding sites of 29 different species. (PDF)
