# Supplementary figures and images for: Lab-on-a-Chip-Based PCR-RFLP Assay for the Detection of Malayan Box Turtle (Cuora amboinensis) in the Food Chain and Traditional Chinese Medicines
Source: PLoS One. 2016 Oct 7;11(10):e0163436. doi: 10.1371/journal.pone.0163436 (PMC5055339; doi:10.1371/journal.pone.0163436)

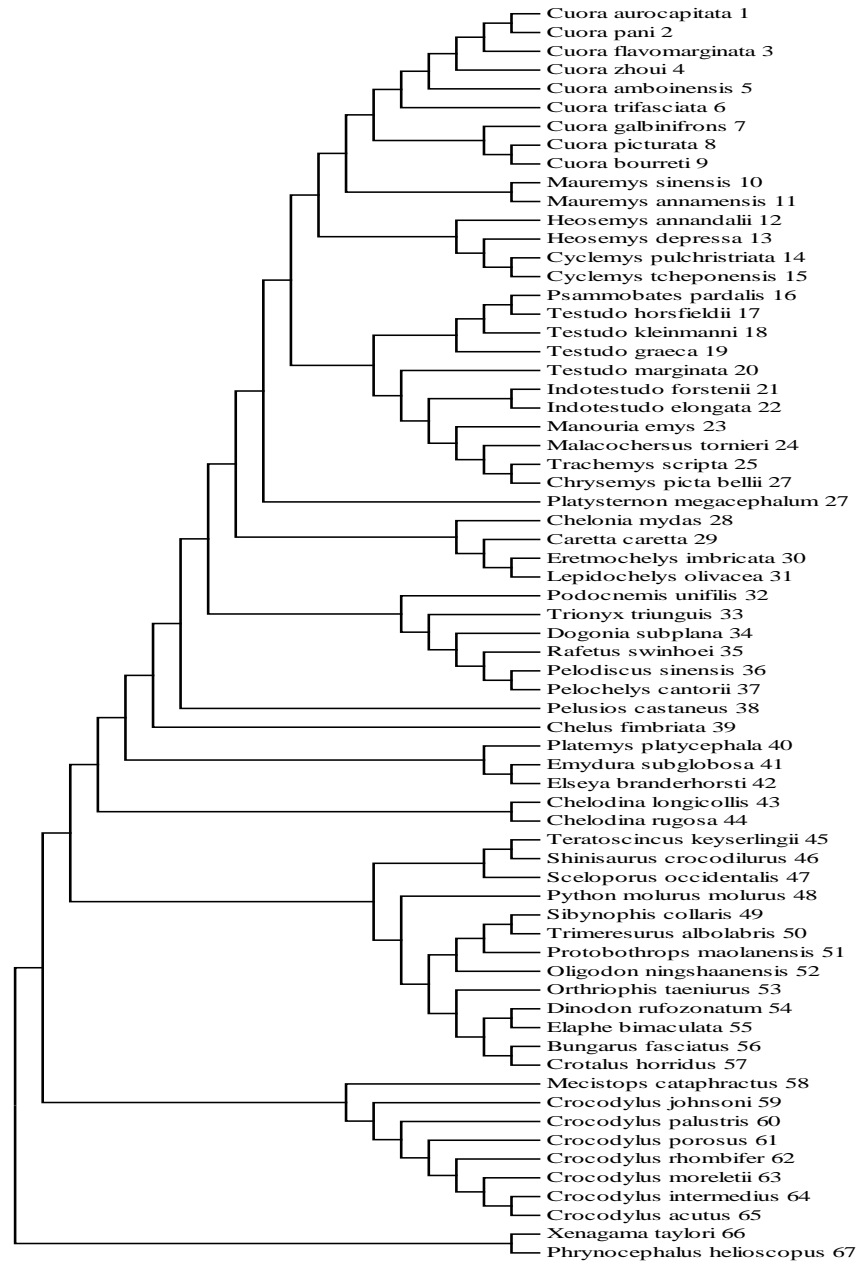

Supplement: S1 Fig — 1–44: turtles/tortoises; 48–57: snakes; 58–65: crocodiles; and 45–47 and 66–67: lizards. Phylogenetic tree was constructed using the Malayan box turtle-specific 120-bp site of the cytochrome b gene against 66 reptile species, of which 44 were turtle (1–44), 10 were snake (48–57), 8 were crocodile (58–65) and 5 were lizard species (45–47 and 66–67). Whereas a very narrow genetic gap was found among the 9 species of the Cuora genus, a wide genetic distance distinguished MBT from the other reptile species. (PDF) [file pone.0163436.s001.pdf]
