# Supplementary material for: Lab-on-a-Chip-Based PCR-RFLP Assay for the Detection of Malayan Box Turtle (Cuora amboinensis) in the Food Chain and Traditional Chinese Medicines
Source: PLoS One. 2016 Oct 7;11(10):e0163436. doi: 10.1371/journal.pone.0163436 (PMC5055339; doi:10.1371/journal.pone.0163436)
Supplement: S1 Table — (PDF) [file pone.0163436.s002.pdf]

	MBT	PST	CBT	YHBT	PBT	ZBT	VBT	INDCBT	BBT	CTSBT	Chicken	Cow	Goat	Pig	Pigeon	Monkey	Rat	Dog	Sheep	Cat	Duck	Buffalo	Deer	Prawn	Cod	Salmon	Carp	Cucumber	Wheat
MBT	0.00																												
PST	0.20																												
CBT	0.11	0.20																											
YHBT	0.09	0.18	0.05																										
PBT	0.09	0.18	0.05	0.00																									
ZBT	0.06	0.17	0.04	0.03	0.03																								
VBT	0.09	0.18	0.09	0.07	0.07	0.04																							
INDCBT	0.09	0.19	0.10	0.08	0.08	0.05	0.04																						
BBT	0.09	0.18	0.09	0.07	0.07	0.04	0.00	0.04																					
CTSBT	0.07	0.16	0.07	0.05	0.05	0.03	0.03	0.04	0.03																				
Chicken	0.44	0.37	0.33	0.33	0.33	0.36	0.39	0.41	0.39	0.36																			
Cow	0.38	0.38	0.39	0.34	0.34	0.34	0.33	0.34	0.33	0.32	0.29																		
Goat	0.41	0.34	0.44	0.37	0.37	0.37	0.33	0.36	0.33	0.34	0.37	0.11																	
Pig	0.37	0.34	0.39	0.34	0.34	0.37	0.36	0.36	0.36	0.33	0.33	0.13	0.16																
Pigeon	0.50	0.40	0.52	0.50	0.50	0.49	0.46	0.49	0.46	0.44	0.31	0.44	0.39	0.38															
Monkey	0.42	0.48	0.39	0.35	0.35	0.35	0.40	0.36	0.40	0.35	0.36	0.32	0.38	0.39	0.59														
Rat	0.41	0.42	0.40	0.35	0.35	0.35	0.36	0.37	0.36	0.35	0.32	0.16	0.16	0.22	0.41	0.39													
Dog	0.44	0.39	0.44	0.39	0.39	0.40	0.37	0.41	0.37	0.40	0.34	0.32	0.32	0.32	0.43	0.52	0.30												
Sheep	0.45	0.43	0.46	0.41	0.41	0.41	0.37	0.38	0.37	0.38	0.42	0.16	0.14	0.19	0.41	0.42	0.23	0.29											
Cat	0.41	0.36	0.35	0.34	0.34	0.31	0.34	0.35	0.34	0.32	0.26	0.19	0.20	0.19	0.44	0.31	0.21	0.33	0.29										
Duck	0.42	0.40	0.40	0.41	0.41	0.38	0.37	0.40	0.37	0.34	0.25	0.39	0.44	0.37	0.36	0.42	0.43	0.40	0.44	0.32									
Buffalo	0.37	0.35	0.38	0.32	0.32	0.32	0.30	0.33	0.30	0.29	0.30	0.09	0.12	0.13	0.38	0.38	0.18	0.35	0.13	0.19	0.37								
Deer	0.44	0.40	0.48	0.41	0.41	0.41	0.37	0.37	0.37	0.38	0.39	0.12	0.13	0.20	0.44	0.42	0.20	0.30	0.14	0.23	0.47	0.13							
Prawn	0.43	0.49	0.53	0.48	0.48	0.48	0.48	0.46	0.48	0.48	0.51	0.47	0.47	0.50	0.57	0.64	0.45	0.50	0.45	0.55	0.61	0.46	0.52						
Cod	0.53	0.44	0.49	0.50	0.50	0.50	0.47	0.47	0.47	0.50	0.38	0.36	0.34	0.34	0.40	0.50	0.36	0.39	0.26	0.33	0.45	0.32	0.31	0.43					
Salmon	0.45	0.37	0.41	0.35	0.35	0.38	0.38	0.41	0.38	0.39	0.35	0.34	0.32	0.31	0.48	0.47	0.32	0.44	0.38	0.29	0.37	0.25	0.36	0.55	0.30				
Carp	0.44	0.34	0.45	0.37	0.37	0.37	0.39	0.41	0.39	0.39	0.31	0.27	0.24	0.30	0.38	0.42	0.28	0.33	0.26	0.27	0.42	0.24	0.30	0.50	0.22	0.21			
Cucumber	0.74	0.67	0.76	0.74	0.74	0.76	0.76	0.78	0.76	0.78	0.72	0.75	0.73	0.69	0.76	0.81	0.78	0.64	0.70	0.80	0.82	0.80	0.71	0.60	0.70	0.73	0.80		
Wheat	0.71	0.64	0.75	0.71	0.71	0.75	0.73	0.75	0.73	0.75	0.74	0.75	0.73	0.69	0.74	0.84	0.78	0.64	0.70	0.83	0.82	0.80	0.71	0.60	0.75	0.78	0.86	0.03	0.00

← 8 species of *Cuora* genus
